# Socioeconomic disparities and the genomic landscape of gastric cancer

**DOI:** 10.1038/s41598-024-65912-6

**Published:** 2024-07-02

**Authors:** Daniel Zanabria, Marco Galvez-Nino, Jhajaira M. Araujo, Alejandro Alfaro, Williams Fajardo, Luis Saravia, Lidia Quispe, Gina Velazque, Junior Carbajal, María J. López, Sergio Jimenez, Paola Montenegro, Alejandra Zevallos, Maria de los Angeles Clavo, Paula Medina-Pérez, Melanie Cornejo, María Requena, Alfredo Aguilar, Joseph A. Pinto

**Affiliations:** 1 Departamento de Patología, Oncosalud-Auna, Lima, Peru; 2 Departamento de Oncología Médica, Oncosalud-Auna, Lima, Peru; 3 Centro de Investigación Básica y Traslacional, Auna Ideas, Av. Guardia Civil 571, San Borja, 15036 Lima, Peru; 4 Servicio de Anatomía Patológica, Hospital Nacional Dos de Mayo, Lima, Peru; 5 Escuela Profesional de Medicina Humana, Universidad Privada San Juan Bautista, Lima, Peru; 6 Servicio de Emergencia, Hospital Regional de Ica, Ica, Peru; 7 Escuela Profesional de Medicina Humana, Universidad Privada San Juan Bautista, Filial Ica, Ica, Peru; 8 Departamento de Patología, Hospital Regional de Ica, Ica, Peru; 9 Servicio de Gastroenterología, Hospital Regional de Ica, Ica, Peru; 10 Facultad de Ciencias Biológicas, Universidad Nacional San Luis Gonzaga, Ica, Peru; 11 Facultad de Ciencias Naturales y Matematicas, Universidad Nacional Federico Villarreal, Lima, Peru; 12 Facultad de Medicina Humana, Universidad Nacional San Luis Gonzaga, Ica, Peru

**Keywords:** Gastric cancer, Targeted therapy, Immunotherapy, Next-generation sequencing, Socioeconomic status, Cancer, Cancer genomics, Cancer therapy, Cancer genetics, Genetic markers

## Abstract

The genomic characteristics of Peruvian patients with gastric adenocarcinoma from diverse socioeconomic backgrounds were examined in consideration of the possibility that patients from different socioeconomic backgrounds may be exposed to different risk factors. We conducted a prospective pilot study in two Peruvian cities (Lima and Ica). This study enrolled 15 patients from low socioeconomic status (LSES) and 15 patients from medium/high socioeconomic status (MHSES). The genomic profiling of gastric adenocarcinoma samples was done through the FoundationOne CDx platform. We compared the genomic characteristics and the need for targeted therapy and immunotherapy between LSES and MHSES. The genes with higher rates of alterations were *TP53* (73.3% vs. 50.0%, *P* = 0.2635); *CDH1* (26.7% vs. 28.6%, *P* = 1); *CDKN2A* (20.0% vs. 28.6%, P = 1); *KRAS* (33.3% vs. 7.1%, *P* = 0.1686); *ARID1A* (20.0% vs. 14.3%, *P* = 1); *MLL2* (13.3% vs. 21.4%, *P* = 1) and *SOX9* (33.3% vs. 0.0%, *P* = 0.0421) in LSES versus HMSES, respectively. There was no significant difference in tumor mutational burden (*P* = 0.377) or microsatellite status (*P* = 1). The LSES group had a higher need for targeted therapy or immunotherapy according to gene involvement and alterations. A significant genomic difference exists among patients with gastric adenocarcinoma of different socioeconomic status, which may result in a different need for targeted therapy and immunotherapy.

## Introduction

Gastric cancer (GC) is the fifth most frequent neoplasm worldwide with an incidence of 11.1 new cases per 100,000 inhabitants which in 2020 caused the death of approximately 780 thousand patients. In Peru, 15.2 per 100,000 new cases are reported each year, being the third most frequent neoplasm and the first cause of death from cancer (≈5000 deaths per year)^1^.

GC is a malignancy with a multifactorial origin, including both environmental and genetic components. Among the identified risk factors are low socioeconomic status, pernicious anemia, family history, high-fat and high-salt diets, smoking, alcohol consumption, and infection with *Helicobacter pylori* (*H. pylori*) or Epstein-Barr virus (EBV)^2–9^. *H. pylori* is the main cause for the high burden of gastric cancer in developing countries, such as Peru. It is also complicated by high antibiotic resistance and lack of treatment adherence in infected patients^8^.

Socioeconomic status has been shown to have a relevance in the incidence of GC. The epidemiology of this malignancy shows that there is a greater incidence of GC in developing countries^7^. Interestingly, a study revealed that the incidence of gastric cancer in the population of affiliates of Oncosalud AUNA (an oncology prepayment system in Peru, with affiliates belonging mainly to socioeconomic sectors A and B), is lower than the incidence reported in metropolitan Lima or the Peruvian population at large^8^. One of the main reasons why a low socioeconomic status influences the risk of GC is the exposure to risk factors, due to poor sanitation conditions and limited access to healthcare services and consequently a more severe evolution of chronic stomach conditions^7^.

In recent years, the prevalence of GC has decreased worldwide. However, it continues to present significant healthcare challenges in developing countries^10^. In this sense, in countries like Peru, prevention and timely diagnosis are important for adequate control of this cancer. Accurately recognizing the risk factors and underlying causes of this disease will contribute to better prevention and subsequent reduction in mortality rates^11^.

We hypothesized that patients with different socioeconomic status have different exposure to contributing risk factors, and therefore differences in genetic lesions could be expected. For this reason, we aimed to compare the influence of the socioeconomic level on the genomic landscape of gastric adenocarcinoma.

## Results

### Characteristics of patients

**Figure 1:**
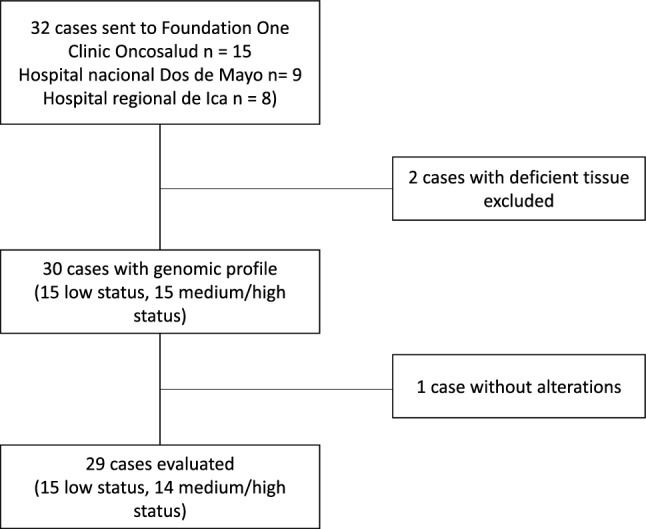
Flowchart of the patients included in the study. Criteria for patients’ selection from three different hospitals (Oncosalud Clinic, Hospital Nacional Dos de Mayo, and Hospital Regional de Ica.

In total, 32 patients met the inclusion criteria, 15 patients were recruited from the Oncosalud Clinic, 9 patients were recruited from the Hospital Nacional Dos de Mayo, and 8 patients from the Hospital Regional de Ica. Two patients were excluded from statistical analysis, as the tissue sample was not sufficient for genomic profiling. Furthermore, a patient was excluded from the bioinformatic analysis due to the absence of pathogenic or likely pathogenic mutations. Finally, 14 patients with low socioeconomic status (LSES) and 15 patients with medium/high socioeconomic status (MHSES) were included for final analysis (Fig. 1). Most of the patients in the LSES group were males (66.7%) and had a median age of 63.0 years of age. On the other hand, patients median age in the MHSES group was 61.0 and most of them were females (53.3%). In addition, the majority were over 65 years old (53.3%) (Table 1).

**Table 1:** Comparison of clinicopathological characteristics between GC with MHSES and LSES.

Characteristics	All patients (n = 30) n (%)	Socioeconomic status		*p* value
		MHSES (n = 15) n (%)	LSES (n = 15) n (%)	
Age (years)^a^	62.5 (47.0–77.8)	61.0 (46.0–76.0)	63.0 (53.5–79.0)	0.575^b^
< 65	16 (53.3%)	8 (53.3%)	8 (53.3%)	1^c^
≥ 65	14 (46.7%)	7 (46.7%)	7 (46.7%)	
Sex				0.272^c^
Women	13 (43.3%)	8 (53.3%)	5 (33.3%)	
Men	17 (56.7%)	7 (46.7%)	10 (66.7%)	
Albumin (g/dL)^a^	3.4 (3.0–4.1)	4.1 (3.9–4.3)	3.0 (2.5–3.3)	< 0.001^b^
Hemoglobin (g/dL)^a^	10.3 (6.6–11.2)	11.2 (10.6–12.5)	7.8 (5.7–9.6)	< 0.001^b^
Tumor size (mm)^a^	35.0 (15.0–57.5)	40.0 (25.5–76.5)	30.0 (12.5–45.0)	0.114^b^
Lauren classification				1^c^
Mixed	3 (10.0%)	2 (13.3%)	1 (6.7%)	
Intestinal	17 (56.7%)	8 (53.3%)	9 (60.0%)	
Diffuse	10 (33.3%)	5 (33.3%)	5 (33.3%)	
Borrmann classification				0.651^c^
I–II	6 (20.0%)	4 (26.7%)	2 (13.3%)	
III–IV	24 (80.0%)	11 (73.3%)	13 (86.7%)	
Location of tumor				0.044^c^
Body-Antrum	2 (6.7%)	2 (13.3%)	0 (0.0%)	
Antrum	13 (43.3%)	4 (26.7%)	9 (60.0%)	
Body	11 (36.7%)	5 (33.3%)	6 (40.0%)	
Fundus	4 (13.3%)	4 (26.7%)	0 (0.0%)	
Clinical stage				0.597^c^
I–II	4 (13.3%)	3 (20.0%)	1 (6.7%)	
III–IV	26 (86.7%)	12 (80.0%)	14 (93.3%)	
Microsatellite status				1^c^
MS-Stable	23 (88.5%)	12 (92.3%)	11 (85.0%)	
MSI-High	3 (11.5%)	1 (7.7%)	2 (15.0%)	
No determined	4	2	2	
Tumor mutational burden (Muts/Mb)^a^	5.0 (3.0–8.0)	3.0 (3.0–5.0)	6.0 (3.0–8.0)	0.377^b^
TMB by groups				0.371^c^
High (≥ 20 Muts/mb)	3 (11.5%)	1 (7.7%)	2 (15.4%)	
Medium (6–19 Muts/mb)	7 (26.9%)	2 (15.4%)	5 (38.5%)	
Low (1–5 Muts/mb)	16 (61,5%)	10 (76.9%)	6 (46.1%)	
No determined	4	2	2	

Comparison of age, sex, clinicopathological variables at diagnosis, microsatellite status and tumor mutational burden (TMB) between low socioeconomic status (LSES) and medium/high socioeconomic status (MHSES) groups.

^a^Median (IQR).

^b^Wilcoxon rank sum test.

^c^Pearson’s Chi-squared test; Fisher’s exact test.

Regarding the Clinicopathological variables at diagnosis (Table 1), we observed that cases with a LSES had lower albumin levels in blood than MHSES [2.9 g/dL (SD ± 0.6) vs. 4.1 g/dL (SD ± 0.9)]; similarly, LSES had inferior hemoglobin levels when compared to MHSES [(7.8 g/dL (SD ± 2.3) vs. 11.2 g/dL (SD ± 1.7)]; all results being of statistical significance.

Furthermore, tumor size was larger in the MHSES group [48.3 mm (SD ± 28.1)]. Histologically, most of the patients in both groups presented the intestinal subtype, according to Lauren classification. Likewise, most of the tumors in both groups were type III-IV according to the Bormann classification.

On the other hand, we must highlight that while LSES patients presented tumors mainly in the antrum of the stomach (60.0%), the MHSES patients showed a similar distribution among the antrum (26.7%), body (33.3%), fundus (26.7%) and body- antrum (13.3%). Finally, more than half of the patients in both groups presented a III-IV clinical stage at diagnosis (93.3% vs. 80.0%, for LSES and MHSES, respectively).

### Comparison of genetic alterations between LSES and MHSES patients with GC

**Figure 2:**
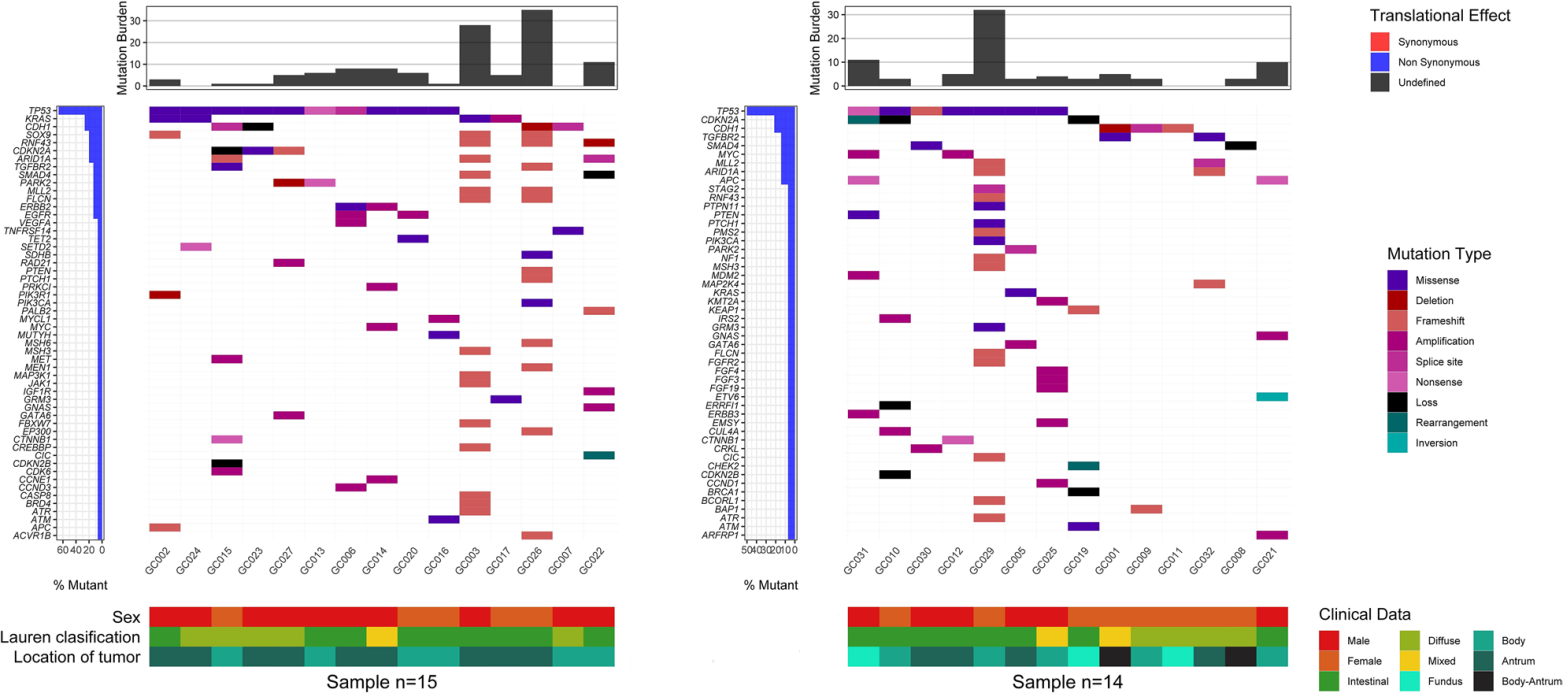
Comprehensive visualization of the genomic landscape of gastric adenocarcinoma in patients participating in this study, (**Left**) Low socioeconomic status and (**Right**) Medium/High Socioeconomic status. We analyzed 324 genes for all patients and found 79 anomalous events. 20.1% of the mutations detected were copy number amplifications, 3.1% were deletions, 5.7% were nonsense mutations, 5.7% were splice-site mutations, 32.1% were frameshift mutations, 24.5% missense mutations, 6.3% loss mutations, 1.9% were genomic segment rearrangements, and 0.6% were inversion mutations.

**Figure 3:**
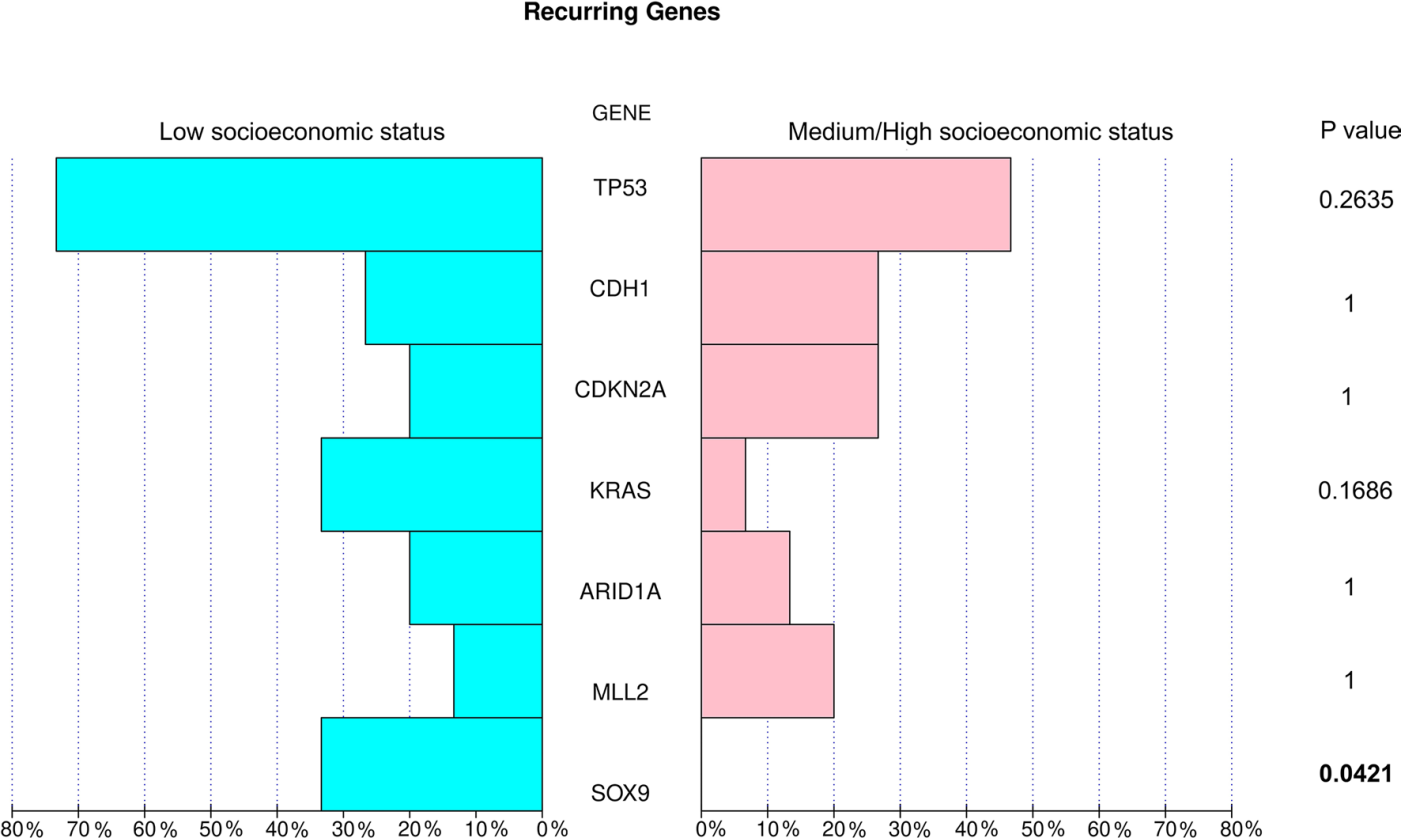
Comparative analysis of mutation between SE groups in genes more frequently altered. In both LSES and HSES, the most frequently mutated genes were *TP53*,* CDH1*,* CDKN2A*,* KRAS*,* ARID1A*,* MLL2*, and *SOX9*. Interestingly, the LSES group presented a higher percentage of mutations in all cases when compared with the MHSES group. In both groups, *SOX9* was the only gene that presented a statistically significant difference (*p* = 0.0421).

**Figure 4:**
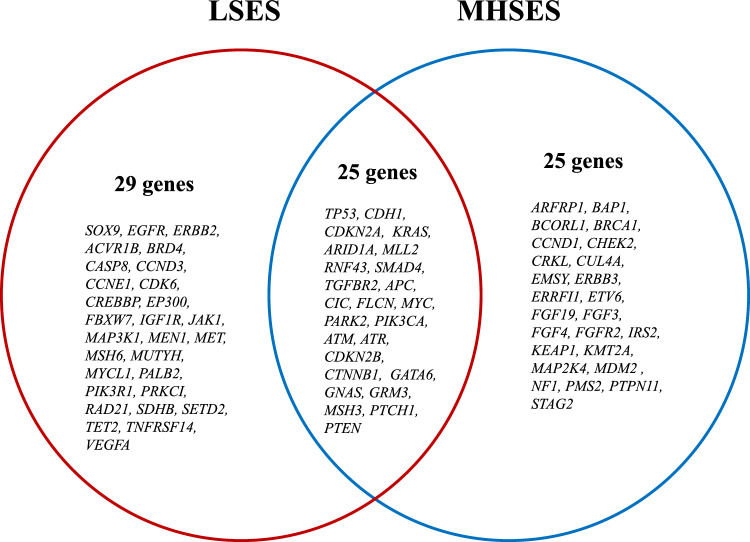
Venn diagram of frequently mutated genes comparing LSES versus MHSES groups. There was a total of 25 genes that presented high mutation rates in both the LSES and MHSES groups.

A total of 324 genes were analyzed for all patients and 79 aberrant events were observed. Additionally, 5.4% (n = 2) of samples harbored only one altered gene. Among the mutations detected, 20.1% presented copy number amplifications, 3.1% copy number deletions, 5.7% nonsense variants, 5.7% splice-site variants, 32.1% frameshift variants, 24.5% missense variants, 6.3% loss variants, 1.9% rearrangement of genomic segments and 0.6% inversion variants (Fig. 2). The most frequently mutated genes in both groups were *TP53* (73.3% vs. 50.0%); *CDH1* (26.7% vs. 28.6%); *CDKN2A* (20.0% vs. 28.6%); *KRAS* (33.3% vs. 7.1%); *ARID1A* (20.0% vs. 14.3%); *MLL2* (13.3% vs. 21.4%) and *SOX9* (33.3% vs. 0.0%) in LSES and HSES, respectively. Interestingly, in all the cases, the LSES group presented a higher percentage of mutations than the MHSES group. The only gene that presented a statistically significant difference between both groups was *SOX9* (*p* = 0.0421) (Fig. 3). In total, 25 genes presented high mutation rates for LSES and MHSES groups (Fig. 4).

**Figure 5:**
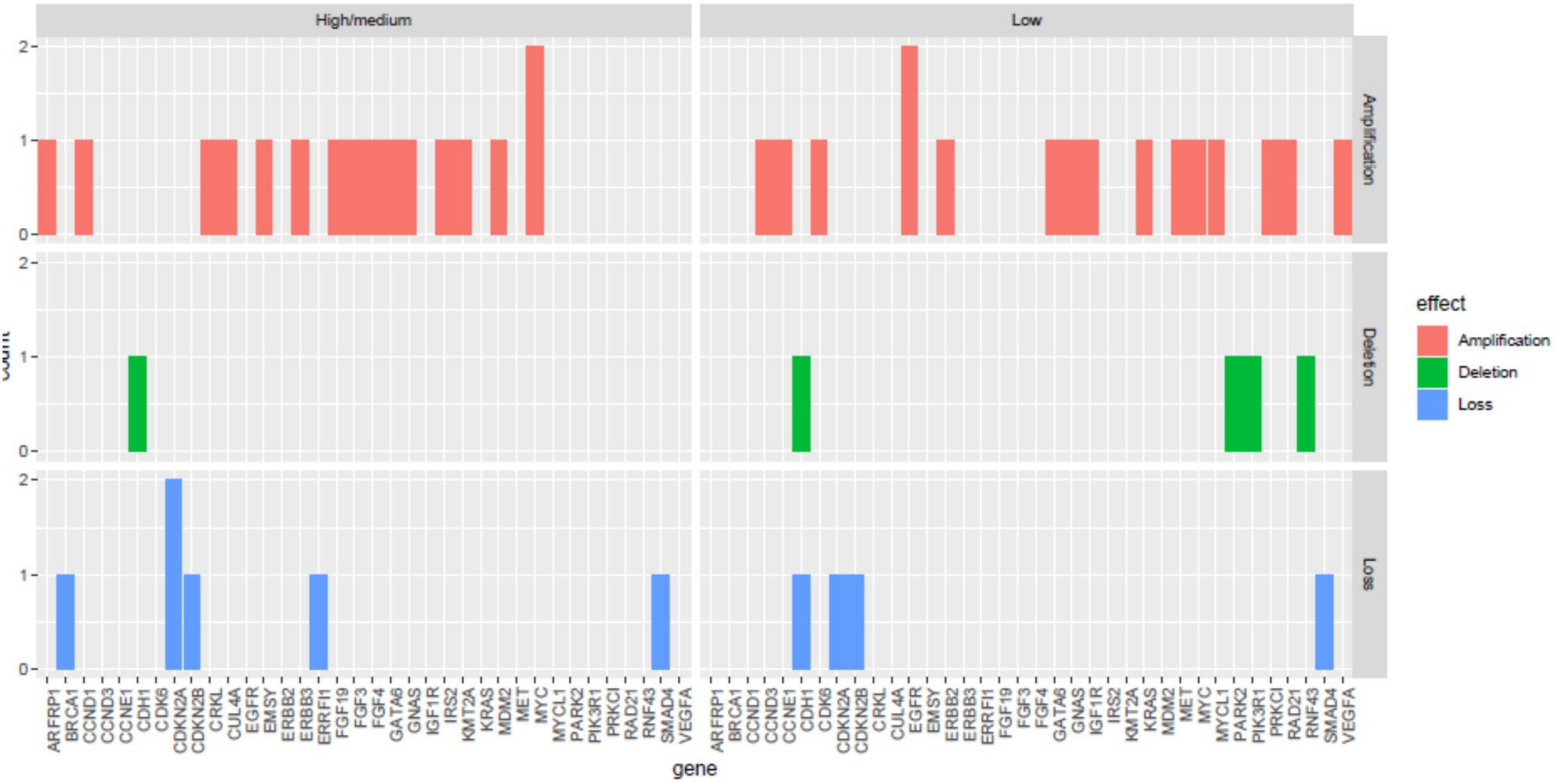
Distribution of genomic alterations among LSES and MHSES patients with gastric adenocarcinoma. The red color indicates copy number amplification. The green color indicates copy number deletion. The blue color indicates copy number loss. According to copy number variation (CNV) analysis, both SE groups displayed similar levels of CNV with amplification. Interestingly, CNVs with amplification were more frequently found in the *MYC* gene in MHSES patients, whereas *EGFR* mutations were more common in LSES patients. MHSES patients exhibited deletions only in the *CDH1* gene, while LSES patients displayed deletions in *CDH1*,* PARK2*,* PIK3R1* and *RNF43* genes. MHSES patients were more likely to demonstrate CNV loss than LSES patients (n = 6 vs. n = 4), with both groups showing mutations in *CDKN2A*,* CDKN2B*, and *SMAD4* genes.

On the other hand, copy number variation (CNV) analysis revealed that both SE groups had similar rates of CNV with amplification. However, it is interesting to highlight that CNV with amplification were more frequently found in the *MYC* gene among MHSES patients, while *EGFR* mutations were more commonly observed in LSES patients. Likewise, MHSES patients only presented CNV with deletion in the *CDH1* gene, while LSES patients presented deletions in *CDH1*, *PARK2*,* PIK3R1 and RNF43* genes. Finally, the CNV loss was found more frequently in MHSES patients than in LSES ones (n = 6 vs. n = 4) with both groups presenting mutations in *CDKN2A*,* CDKN2B* and *SMAD4* (Fig. 5).

We can observe that most patients in the LSES and MHSES groups had a stable microsatellite status (85.0% vs. 92.3%). However, the proportion of patients with a high microsatellite status was higher in the LSES group than in the MHSES group (15.4% vs. 7.7%). Regarding tumor mutational burden (TMB), the mean was higher in the LSES group [9.1 mut/Mb (SD ± 10.5)]. Interestingly, a low TMB was dominant in both groups (LSES and MHSES) (46.1% and 76.9%) (Table 1).

### Molecular profiling for rational selection of targeted and immunotherapies

**Figure 6:**
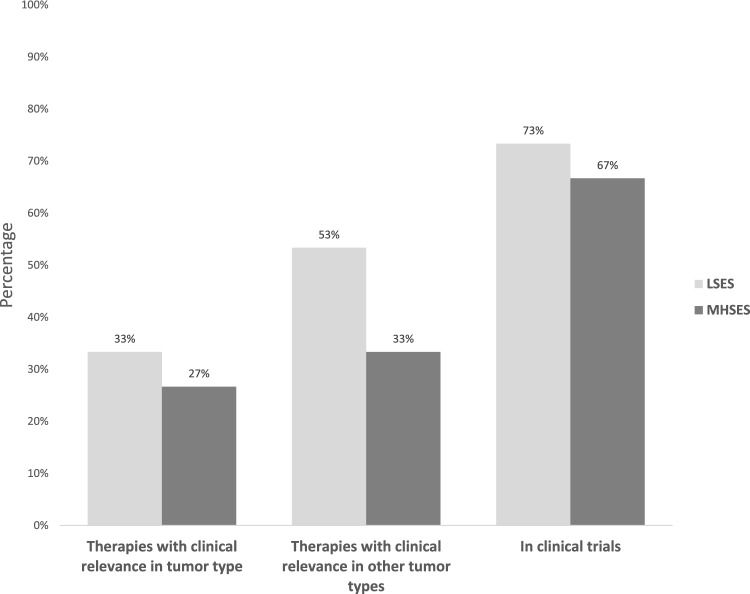
Distribution of therapies with clinical benefit between LSES and MHSES groups. Overall, LSES patients had a higher potential inclusion rate in clinical trials than MHSES patients (73.0% vs. 67.0%). Accordingly, the percentage of patients who would qualify for drug treatment with clinical relevance in other tumor types was higher in the LSES group than in the MHSES group (53% vs. 33%).

Based on the data provided by the genomic profiling reports, we organized potential therapies for each SES group (Tables 2 and 3). Thus, 33.0% of patients within the LSES group presented mutations that literature has already shown as druggable, with clinical relevance in gastric cancer, while for MHSES, only 27.0% of patients were adequate for these treatments. Similarly, the percentage of patients who would be good candidates for drugs with clinical relevance in other tumor types was higher in the LSES group than in the MHSES group (53.0% vs. 33.0%). In general, patients from the LSES group had a higher percentage of potential inclusion than the MHSES group in clinical trials (73.0% vs. 67.0%) (Fig. 6).

**Table 2:** Candidate target therapy according to genomic findings to gastric adenocarcinoma patients included in this study.

SES	Genomic findings	Drugs with clinical relevance in gastric cancer	Drugs with clinical relevance in other tumor types
LSES	FBXW7-C46fs*14	None	Everolimus, Temsirolimus
	ERBB2-G660D	Fam-trastuzumab deruxtecan, Trastuzumab	Ado-trastuzumab emtansine, Lapatinib, Neratinib, Trastuzumab+Pertuzumab
	EGFR-amplification-equivocal	None	Afatinib, Cetuximab, Dacomitinib, Panitumumab
	ERBB2-amplification	Trastuzumab, Fam-trastuzumab deruxtecan, Trastuzumab+Pembrolizumab	Ado-trastuzumab emtansine, Afatinib, Dacomitinib, Lapatinib, Margetuximab, Neratinib, Trastuzumab+Pertuzumab
	MET-amplification	None	Cabozantinib, Capmatinib, Crizotinib, Tepotinib
	ATM-R337H	None	Niraparib, Olaparib, Rucaparib, Talazoparib
	EGFR-amplification	None	Afatinib, Cetuximab, Dacomitinib, Panitumumab
	PALB2-S288fs*15	None	Niraparib, Olaparib, Rucaparib, Talazoparib
	PIK3CA-Q546R	None	Everolimus, Temsirolimus
MHSES	ATM-R337H	None	Niraparib, Olaparib, Rucaparib, Talazoparib
	BRCA1-loss exons 15–19	None	Niraparib, Olaparib, Rucaparib, Talazoparib
	FGFR2-S799fs*17	None	Erdafitinib
	NF1-I679fs*21	None	Selumetinib, Trametinib
	PIK3CA-H1047R, R93Q	None	Everolimus, Temsirolimus

The table shows potential therapies for each SES group, which were based on patient genomic analysis to determine drugs with clinical relevance in gastric cancer as well as drugs with clinical relevance in other tumor types.

**Table 3:** Candidate immunotherapy according to genomic findings in gastric adenocarcinoma patients included in this study.

SES	Biomarker findings	Drugs with clinical relevance in gastric cancer	Drugs with clinical relevance in other tumor types
LSES	Microsatellite status-MSI-High	Nivolumab, Pembrolizumab, Dostarlimab*	Atezolizumab, Avelumab, Cemiplimab, Dostarlimab*, Durvalumab
	TMB-high	Nivolumab, Pembrolizumab, Dostarlimab*	Atezolizumab, Avelumab, Cemiplimab, Dostarlimab*, Durvalumab, Nivolumab+Ipilimumab
MHSES	Microsatellite status-MSI-High	Nivolumab, Dostarlimab*, Pembrolizumab	Atezolizumab, Avelumab, Cemiplimab, Durvalumab
	TMB-high	Nivolumab, Pembrolizumab, Dostarlimab*	Atezolizumab, Avelumab, Cemiplimab, Durvalumab, Nivolumab, Nivolumab+Ipilimumab

Potential therapies for each SES group, which were based on patient biomarker findings to identify drugs with clinical relevance in gastric cancer, as well as drugs with clinical relevance in other types of cancer.

*FDA-approved for the treatment of solid tumors, including gastric cancers.

## Discussion

Gastric cancer is a malignancy of high incidence and high mortality rates in developing countries. GC patterns could vary between different ethnic groups suggesting differences in genomic alterations, beyond those expected by disparities in cancer care attention^12,13^. For this reason, we conducted a pilot study in Peruvian patients to evaluate if socioeconomic status influences the presentation of genomic alterations in gastric adenocarcinoma, using a comprehensive genomic platform.

Several studies have already demonstrated that gastric cancer incidence and mortality were higher among people of a low socioeconomic status^14–16^. The mechanisms underlying the influence of SES on gastric cancer risk are unclear, however they may reflect differences in access to medical resources^17,18^ and divergence in health behaviors^19,20^.

A metanalysis by Yusefi et al.^16^ showed that a higher educational level leads to a higher consumption of fruits and vegetables and a reduction in smoking, in comparison to a lower educational level. Likewise, *Helicobacter pylori* (*H. pylori*) infection has also been reported to be more common among subjects with lower levels of education^21,22^. Another study that took into consideration patients’ occupation showed that unemployed and manual laborers have a worse prognosis than employed people, leading to disparities in survival rates among gastric cancer patients^23^. A study by Chin-Chia demonstrated that even in patients receiving curative-intent therapy for gastric cancer, patients aged less than 65 years of low SES have the worst outcomes^24^. In addition, previous studies also indicate that uninsured patients are more likely to present with advanced-stage cancers and have a reduced likelihood of receiving definitive therapy^18^.

Regarding the Peruvian population, there are not previous reports comparing different SES. However, there are estimations about the proportion of attributable fraction (PAF) about the main modifiable risk factors for gastric cancer and we could infer some differences according to SES. For example, the PAF for *Helicobacter pylori* (mainly present in LSES) is 46.4% while red meat consumption and processed meat consumption (more prevalent in MHSES) is 18.9% and 6,2%, respectively^25^.

The considerable variation of the disease between racial groups has led to a substantial knowledge gap in the treatment of patients. In our study, LSES patients presented tumors mainly in the antrum of the stomach (60.0%), while MHSES patients showed a similar distribution among the antrum (26.7%), body (33.3%) and fundus (26.7%). Several studies show that Hispanic-Americans are more likely than non-Hispanic Whites to develop gastric adenocarcinoma, especially non cardia gastric cancers^26–31^. Furthermore, a study by Kim et al.^32^ which considered patients with gastric cancer from Los Angeles County showed that Asians, Hispanics, and Blacks presented the lowest incidence of cardia tumors, while the highest incidence was found in Whites.

In our study, most of the tumors in both SE groups presented with more advanced AJCC stage disease (stage III or IV) which goes in accordance with the literature. A study by Al-Refaie et al.^12^, which used the National Cancer Data Base (NCDB), demonstrated that African Americans and Hispanics tend to present advanced clinical stages. Likewise, it showed that grade 3 histologic differentiation was more prevalent in Hispanics and Asian Pacific Islanders than in whites, African Americans, and others (*P* < 0.01). In addition, a study by Tseng et al.^33^ reported that Hispanic and Black patients are more likely to suffer from aggressive diseases and to be plagued by financial problems which make their overall survival less likely.

Regarding genomic alterations, *TP53*,* CDH1*,* CDKN2A*,* KRAS*,* ARID1A*,* MLL2* and *SOX9* were the most frequently mutated genes. Interestingly, in all the cases, the LSES group presented a higher percentage of mutations than the MHSE group. According to the literature, it is recurrent for GI cancers to have mutations in *TP53*,* APC*,* KRAS*,* BRAF*, and *PIK3CA*^34^; however, mutation frequencies vary depending on tumor type^35^. Notably, another study conducted on a Latin-American population identified the principal somatic variants in Mexican patients with gastric adenocarcinoma also being *TP53*,* CDH1*,* CDKN2A* and *ARID1A*^36^. In addition, *CDH1* and *KMT2C* have been previously linked to diffuse-type GA^37,38^.

*SOX9* is a gene involved in chondrogenesis, while its inactivation during embryonic development leads to campomelic dysplasia^39^. Recent studies have reported the participation of *SOX9* in the regulation of stem cells, cell plasticity and the epithelial-mesenchymal transition, which promotes the activation of several signaling pathways leading to cancer development, cancer progression, and drug resistance^40^. On the other hand, in gastric cancer, *SOX9* promotes progression and metastasis in gastric cancers by the suppression of CD8+T cell responses^41^. The TCGA project has shown that 6% of gastric cancers had genomic alterations, mainly CNV´s14. In our work, *SOX9* mutations corresponded mainly to frameshift alterations (Fig. 2). *SOX9* is an interesting therapeutic target in cancer. In vitro and in vivo studies have shown that suppression of *SOX9* inhibits proliferation of stomach, lung, and prostate cancer cells^42–44^.

This work has some limitations. Although the groups were similar in most of their clinicopathological characteristics, due to the small sample size and consequently, the lack of statistical power to perform multivariate analyses, it was not possible to evaluate the impact of some possible confounding factors. Besides, we could not include all variables associated to genomic alterations and socioeconomic status, such as obesity or diabetes^45–47^. The limited sample size may have also affected the power to detect some differences in genomic alterations. However, this pilot study is a strong foundation for further studies, as we used a robust and validated technology to evaluate genomic alterations. To our knowledge, it is the first study to evaluate the influence of disparities in the genomic landscape of gastric cancer and it shows an interesting therapeutic landscape for gastric cancer. The current FDA-approved therapeutics for gastric cancer include trastuzumab, entrectinib, larotrectinib, selpercatinib, dabrafenib, trametinib, pembrolizumab and nivolumab.

NGS technology and interpretation of results is becoming more common. A 2018 US study showed that 75% of physicians are confident that genomic testing can improve patient outcomes and about 50% are confident in their ability to interpret molecular test results. However, there are concerns about affordability and accessibility: the cost of sequencing in low-income countries can be five times higher than in high-income countries due to taxes and the high cost of testing, shipping, and infrastructure^48^. Currently, ERBB2 and MMR analysis can be performed by IHC to be able to use approved therapies; however, it is expected that the use of NGS will become an important tool for both diagnosis and treatment as occurs in other solid tumors, since it will allow the identification of future actionable alterations and biomarkers of response or resistance^49^. NGS may enable the integration of molecular tumor profiling into clinical decision-making as part of precision oncology^50^.

In conclusion, our exploratory study suggests differences in the genomics of gastric adenocarcinoma between different socioeconomic status and a potentially increased need for targeted therapy and immunotherapy in LSES. On the other hand, it shows the potential of NGS sequencing for gastric cancer diagnosis and treatment, and the challenges of its implementation in low resource settings. Further studies with larger sample sizes are necessary to deepen our understanding of the influence of socioeconomic status on the genesis and evolution of different types of cancers. Although, our results should be evaluated carefully, these findings could indicate an upcoming public health challenge, as new therapies are approved for gastric cancer.

## Methods

### Study design

We conducted a prospective, exploratory, observational, hypothesis-generating, case-control study that included patients with gastric cancer treated at two public hospitals (Hospital Nacional Dos de Mayo in Lima and the Hospital Regional de Ica in Ica), and one private clinic (Oncosalud–AUNA).

### Population and eligibility criteria

The study population involved patients with gastric adenocarcinoma diagnosed between 2020 and 2022. Inclusion criteria were, being 18 years or older, previously untreated, adequate stored histological material, and acceptance of participation in the study by signing an informed consent. Patients with an unsuitable tumor sample for genomic analysis were excluded.

### Tumor sample collection

Gastric biopsies and surgical pieces of gastric tumors were prospectively collected. The samples were fixed in formalin, embedded in paraffin and histological sections were cut. An expert pathologist oversaw the evaluation of the hematoxylin and eosin-stained slides. The paraffin-embedded tissues were sent to Foundation Medicine (Cambridge, US) for analysis through the FoundationOne CDx platform.

### Collection of clinicopathological information

Clinical records from each patient were reviewed. The clinicopathological variables included age, sex, histological classification, Lauren’s classification, Borrmann’s classification, tumor size and clinical stage.

### Data collection instruments

For the socioeconomic status (SES) evaluation, patients were interviewed using a sociodemographic questionnaire developed by The Peruvian Association of Market Intelligence Companies (APEIM)^51^, which classifies SES into five levels based on the scores they obtain on the questionnaire (A: 40 points or more, B: From 29 to 39 points, C: From 20 to 28 points, D: From 13 to 19 points and E: 12 points or less). For this study, patients from levels A, B, and C were classified as MHSES, while patients from levels D and E were classified as LSES.

### Genomic analysis

The Foundation One CDx platform includes analysis of point mutations and copy number variation in 324 driver genes; as well as the tumor mutational load (# per 1000 bases); microsatellites instability and, genetic rearrangements in 32 driver genes: *BCL2*,* BCR*,* BRAF*,* BRCA1*,* BRCA2*,* CD74*,* EGFR*,* ETV4*,* ETV5*,* ETV6*,* EWSR1*,* EZR*,* FGFR1*,* FGFR2*,* FGFR3*,* KIT*,* KMT2A*,* (MLL)*,* MSH2*,* MYB*,* MYC*,* NOTCH2*,* NTRK1*,* NTRK2*,* NUTM1*,* PDGFRA*,* RAF1*,* RARA*,* RET*,* ROS1*,* RSPO2*,* SDC4*,* SLC34A2*, TERC*,* TERT* and *TMPRSS2*.

### Evaluation of suitable targeted therapies and immunotherapy

We grouped clinical therapies according to reports provided by Foundation One CDx into three trials for each socioeconomic level, clinically relevant therapies in the same tumor, clinically relevant therapies in another tumor type, and clinical trials.

### Statistical analysis

The primary study endpoint was differences in genomic alterations, the secondary endpoint was differences in clinic pathological characteristics and the potential use of targeted and immune therapy between SES.

Descriptive statistics included frequencies for categorical data and mean, median, and standard deviation for quantitative data. We used graphs to explain the results of the numerical variables and descriptive statistics, including frequencies for categorical variables. For the calculation of the quantitative variables, we used the Wilcoxon test, while for the qualitative variables the Fisher exact test was used. The GenVisR package, ggplot2^52^, was used to visualize genomic alterations. Alterations of each individual gene were visualized using the MutationMapper platform on the cBioPortal website^53^. We used a statistical significance of a *p*-value less than 0.05 for all hypothesis tests. Because it was a preliminary study, the comparisons of the hypothesis tests were not adjusted for the *p*-values. The data collected was analyzed in SPSS v.21; in R language v.4.1.2 and RStudio v.1.3.1093-1.

### Ethical disclosure

The study was approved by the Institutional Ethics Committee “Comité Institucional de Bioética en Investigación de VÍA LIBRE”. All procedures performed in the study were in accordance with the ethical standards, relevant guidelines and with the Helsinki declaration. Patients signed an informed consent to participate in the study.

## Data Availability

The datasets presented in this study can be found in online repositories. The names of the repository/repositories and accession number(s) can be found at: 10.6084/m9.figshare.25183367.
